# Comparative profiling of differentially expressed microRNAs between the follicular and luteal phases ovaries of goats

**DOI:** 10.1186/s40064-016-2902-1

**Published:** 2016-08-02

**Authors:** Long Zhu, Tao Chen, Menghua Sui, Chunyang Han, Fugui Fang, Yuehui Ma, Mingxing Chu, Xiaorong Zhang, Cuiyan Liu, Yinghui Ling

**Affiliations:** 1 College of Animal Science and Technology, Anhui Agricultural University, 130 Changjiang west road, Hefei, 230036 Anhui China; 2 Local animal genetic resources conservation and biobreeding laboratory of Anhui province, 130 Changjiang west road, Hefei, 230036 Anhui China; 3 Key Laboratory of Farm Animal Genetic Resources and Germplasm Innovation of Ministry of Agriculture, Chinese Academy of Agricultural Sciences, Institute of Animal Science, 2 Yuanmingyuan West Road, Beijing, 100193 China

**Keywords:** *Capra hircus*, Post-transcriptional, Follicular-luteal transition, Next generation sequencing

## Abstract

To explore if the regulation at post-transcriptional level of follicular phase (Fols) to luteal phase (Luts) transition occurs in the ovaries of Anhuai goats, the differentially expressed microRNAs (miRNAs) of ovaries in the Fols and Luts were analyzed using Solexa sequencing in the study. In total, 320 known miRNAs were co-expressed in the two phases, 339 and 353 known miRNAs were expressed in the ovary in the Fols and Luts, respectively. In addition, 45 novel miRNAs were co-expressed in the two phases, 70 and 94 novel miRNAs were expressed in the ovary in the Fols and Luts, respectively. Let-7f was the highest expressed significantly different known miRNA in the two phases, and mir-159 was the highest expressed significantly different novel miRNA in the two phases, which may participate in the follicular-luteal transition of Anhuai goats. GO annotation and KEGG pathway analysis were applied to analyze the target genes of differentially expressed miRNAs detected in the two phases. The results will help to further understand the role of miRNAs in the regulation of follicular to luteal transition in goat ovaries.

**Electronic supplementary material:**

The online version of this article (doi:10.1186/s40064-016-2902-1) contains supplementary material, which is available to authorized users.

## Background

Ovarian activities are vital to successful reproduction and maintaining reproductive efficiency in mammals. Growth and regression of follicles, ovulation, differentiation of mature follicle cells into luteal cells and growth and subsequent demise of corpora luteal occur in the ovary within the short time frame of a reproductive cycle and occur repeatedly throughout a female’s reproductive life (McBride et al. 2012), meanwhile, he ovarian activities determine the estrous cycle sequence.

It has been reported that the transition of follicular and luteal cell was mainly controlled by follicle-stimulating hormone and luteinizing hormone. Briefly, gonadotropin-releasing hormone (GnRH) is released from the hypothalamus and controls the release of the follicle-stimulating hormone (FSH) and luteinizing hormone (LH) from the pituitary gland which then induce ovarian steroid secretion that impacts the hypothalamus and pituitary through a feedback mechanism (Richards and Pangas 2010a). According to research on specific breeds of mice, the roles of bone morphogenetic proteins (BMPs), activins, Smads, Wnt signaling, and aspects of the FSH and LH signaling cascades are emphasized in the regulation of cyclic ovarian activity (Puglisi et al. 2004; Tomizuka et al. 2008). In addition, the regulation of follicular and luteal cell function in rodents has been described in transcriptional level (Richards 2001; Richards and Pangas 2010b). However, the regulation mechanism of Fols to Luts transition on the post-transcriptional is limited.

microRNAs (miRNAs) are a group of endogenous 18–22 nt small non-coding RNAs that can regulate mRNA degradation at the post-transcriptional level or inhibit mRNA translation to modulate gene expression (Bartel 2004). miRNAs regulation have been implicated in varied physiological processes including tumorigenesis, hormone secretion, differentiation, apoptosis, cell proliferation, metabolism and reproduction control (Ambros 2004; Cuellar and McManus 2005; Hwang and Mendell 2006; Hu et al. 2008; Ivey and Srivastava 2010; Hawkins et al. 2011; Jansson 2012; Rottiers and Näär 2012). Differentially expressed miRNAs can further reveal the mechanism of development of the follicle and luteal, hormone secretion and other reproductive activity in goats.

The function of miRNAs in the mammary gland (Ji et al. 2012), development of hair follicle (Liu et al. 2012) and reproduction or muscle of goat (Ling et al. 2013; Zhang et al. 2013) have been studied in the recent years. It have been showed that GnRH promote the expression of follicle stimulating hormone beta (FSHB) by miR-132/212 and SIRT1-FOXO1 pathway, which was the first miRNA pathway of GnRH regulating the expression of gonadotropin genes (Lannes et al. 2013). Expression of several miRNAs was regulated by FSH, and FSH regulated generation of follicle by miRNA network in the ovary (Yao et al. 2009). Let-7b has been identified in the development of normal luteum which was involved in FSH secretion regulation (Cao et al. 2015). The differential expression of sRNAs in specific stages of the ovine estrous cycle were identified, and identified population of miRNA were specifically associated with follicular to luteal transition in sheep (McBride et al. 2012).

Anhuai goat is a native goat in China, which is known for its higher fertility, precocious puberty, and higher leather quality compared with other goat. The kidding rate of Anhuai goat is 2.27–2.39, which is belonged to varieties of higher kidding rate in goats, and the ewes can estrus all year round (Ling et al. 2014). Therefore, it is an ideal model for the study of goat breeding traits. To investigate the regulation mechanism of Fols to Luts transition of goats on the post-transcriptional level, we characterized and investigated the expression of miRNAs in the ovaries of Anhuai goats in Fols and Luts by deep sequencing technology in the study. In addition, the candidate target genes of all miRNAs in the two phases were predicted, and GO and KEGG pathway analysis of the miRNA target genes were analyzed. The results will help to further understand the regulation at post-transcriptional level of Fols to Luts transition and also may help to identify miRNAs which could be used to shorten the estrous cycle in the future.

## Methods

### Sample preparation

The experimental goats in this study, Anhuai goats (a Chinese indigenous breed), were obtained from an experimental farm (College of Animal Science and Technology, Anhui Agricultural University, Hefei, China). Based on long term observations, laparoscopic and ultrasound detection, the six target goats, three were in follicular phase and the other three were in luteal phase, were chosen as our experimental samples for their ovaries. The physical condition and age of experimental samples were basically consistent. To eliminate the influence of other factors, the unified management system of the field was adopted in the study farm feeding and stabling.

After slaughter, both whole ovaries from each goat were collected and frozen in liquid nitrogen instantly, then stored at −80 °C. The half of the ovaries was used for RNA-Seq, whereas the other half was used for real-time PCR. All experimental procedures received prior approval by the ethics committee of Anhui Agricultural University, Anhui, China (permit no. AHAU20101025 the Regulations for the Administration of Affairs Concerning Experimental Animals, Ministry of Science and Technology, China; revised June 2004).

To minimize differences among individuals, the three whole ovaries from goat in Fols and Luts, respectively, were grouped for RNA extraction using RNAiso Plus (TaKaRa, Japan). Briefly, the correct amount of the frozen tissue sample was dissolved in 1 ml RNAiso Plus. After incubation for 5 min at room temperature, the homogenate was centrifuged at 12,000*g* for 5 min at 4 °C. The supernatant was then transferred to a new centrifuge tube. Chloroform (1/5 volume of RNAiso Plus) was then added to the supernatant. The solution was fully emulsified and incubated for 5 min at room temperature before centrifugation at 12,000*g* for 15 min at 4 °C. The supernatant was then carefully transferred to another centrifuge tube. An equal amount of isopropanol was added to the supernatant. After the solution was fully mixed and incubated for 10 min at room temperature before a further centrifugation at 12,000*g* for 10 min at 4 °C. Then, 1 ml 75 % ethanol was slowly added to the white precipitate, which was centrifuged at 12,000*g* for 5 min at 4 °C. Finally, the supernatant was discarded and the precipitate was dried and dissolved in RNase-free ddH_2_O. The quantity and quality of the total RNA were measured using the Agilent 2100 Bioanalyzer system (Santa Clara, CA) and the samples were stored at −80 °C until analysis.

For library preparation, mRNA was first extracted from total RNA using oligo (dT) magnetic beads and sheared into short fragments of about 200 bases. These fragmented mRNAs were used as templates for cDNA synthesis. The cDNAs were then PCR amplified to complete the library. The cDNA library was sequenced using an Illumina HiSeq™ 2000 platform.

### Raw data cleaning and quality control of sequencing

Small RNA fragments of 18–30 nt in length were isolated and purified from total RNA using 15 % denaturing polyacrylamide gel electrophoresis (PAGE). Subsequently, a 5′ RNA adaptor and 3′ RNA adaptor were ligated to the RNA pool using T4 RNA ligase, the samples were used as templates for cDNA synthesis. The cDNAs were amplified using the appropriate number of PCR cycles to produce sequencing libraries, which were subsequently subjected to the proprietary Solexa sequencing-by-synthesis method using the Illumina Genome Analyzer (SanDiego, CA, USA) at the Beijing Genomics Institute (BGI, Shenzhen, China).The very basic figure from sequencing was converted into sequence data by the base calling step. We evaluated the raw data on the two aspects: filtering contaminants, and initial judgment on the data. The data was processed by the following steps: getting rid of low quality reads: reads with 5′ primer contaminants, without 3′ primer, without the insert tag, with poly A, shorter than 18 nt; statistic of data quality and length distribution after filtering; summarising the common and specific tags of two samples, including the summary of unique tags and total tags.

### Known miRNA screening and novel miRNA prediction

To make every unique small RNA could be mapped to only one annotation, we annotated the sRNA followed the following priority rule: rRNAetc (in which Genbank >Rfam) > known miRNA > piRNA > repeat > exon > intron3. The total rRNA proportion is a mark for sample quality check. It should be less than 60 % in plant samples and 40 % in animal samples as high quality by BGI in generally (Hao et al. 2012).

The clean reads were aligned to the miRNA precursor/mature miRNAs of all animals using miRBase 21.0 (http://www.mirbase.org/), and the sequences and counts of the miRNA families (no specific species) in both groups were displayed. The characteristics of the miRNA precursor hairpin structures were used to predict novel miRNAs. The un-annotated sequences were mapped to the hircine genome to predict potential novel miRNA candidates using Mireap (http://www.sourceforge.net/projects/mireap/).

### Differential expression analysis of miRNA

The expression of known and novel miRNAs was compared between Fols and Luts to found out the differentially expressed miRNAs. The expression of miRNA in f isopropanol was showed by plotting Log2-ratio figure and scatter plot. The expression of miRNA in two samples (Fols was used as a control and Luts was treatment) was normalized to get the expression of transcript per million (TPM). Normalization formula: (1) normalized expression = Actual miRNA count/Total count of clean reads ×1,000,000; (2) Calculate fold-change and p value from the normalized expression. Then generate the log2-ratio plot and scatter plot. Fold-change formula: Fold-change = log2 (treatment/control). The p value formula was:

$$p\left( {y|x} \right) = \left( {\frac{{N_{2} }}{{N_{1} }}} \right)^{y} \frac{{\left( {x + y} \right)!}}{{x!y!\left( {1 + \frac{{N_{2} }}{{N_{1} }}} \right)^{{\left( {x + y + 1} \right)}} }}\quad \begin{array}{*{20}l} {C\left( {y \,\le\, y_{{\rm min} } |x} \right) = \sum\limits_{y \,=\, 0}^{{y\, \le\, y_{{\rm min} } }} {p\left( {y|x} \right)} } \\ {D\left( {y \,\ge\, y_{{\rm max} } |x} \right) = \sum\limits_{{y \,=\, y_{{\rm max} } }}^{\infty } {p\left( {y|x} \right)} } \\ \end{array}$$

The x and y represent the normalized expression level, and the N1 and N2 represent the total count of clean reads of a given miRNA in small RNA library of ovaries of Fols and Luts goats, respectively (Huang et al. 2011). miRNAs’ expression value was revised to 0.01 when the normalized expression of the certain miRNA was zero in one of the two libraries. If the normalized expression of a certain miRNA was all lower than 1 in two libraries, further differential expression analysis was conducted without this miRNA for the reason of its low expression.

### Target genes prediction and function analysis of the differentially expressed miRNA

RNAhybrid software was performed to predict target gene of miRNA in the sequencing. GO enrichment analysis in our study was successful at predicted target gene candidates of all differentially expressed miRNAs compared to the reference gene background, as well as the genes that corresponded to certain biological function. Goseq and topGO will be used to describe the enrichment level for each GO term. This method maps all target gene candidates to GO terms in the database (http://www.geneontology.org/), calculating the gene numbers for each term, then use hypergeometric test to find significantly enriched GO terms in target gene candidates comparing to the reference gene background. We used the Bonferroni Correction for the p value to obtain a corrected p-value. GO terms with corrected p value ≤0.05 are defined as significantly enriched in target gene candidates.

KEGG is the major public pathway-related database. KEGG pathway analysis identified significantly enriched metabolic pathways or signal transduction pathways in target gene candidates of all differentially expressed miRNAs comparing with the whole reference gene background (Kanehisa et al. 2008). The calculating formula was the same as that in the GO analysis. Where N was the number of all genes with a KEGG annotation, n was the number of target gene candidates in N, M was the number of all genes annotated to a certain pathway, and m was the number of target gene candidates in M. Genes with a Q value ≤0.05 were considered as significantly enriched in target gene candidates. The KEGG analysis could reveal the main pathways which the target gene candidates are involved in.

### Real-time PCR of miRNAs

To validate the Solexa sequencing data, real-time PCR (qRT-PCR) assay was carried out. One microgram of total RNA from each sample was reverse transcription into cDNA using the SYBR^®^ Prime Script™ miRNA RT-PCR Kit (TaKaRa, Japan) according to the manufacturer’s protocol. The reverse primer was provided in the kit, and the forward primers were designed based on the mature miRNA sequences (Table 1). After incubation at 37 °C for 1 h and deactivation at 95 °C for 5 min, the template for qRT-PCR was got. The reaction system of qRT-PCR contained 2.0 μl cDNA, 7.5 μl SYBRGreen Mix (Thermo, Shanghai, China), 0.45 μl of each primer and 4.6 μl ddH_2_O. qRT-PCR was performed using standard protocols on the StepOnePlus™ Real-Time PCR System (Thermo Fisher Scientific, American). The reaction was incubated at 95 °C for 10 min, followed by 40 cycles of 95 °C 15 s, 60 °C 60 s. All reactions were performed in triplicate, and the reaction solution was prepared on ice. The threshold cycle (CT) was collected from each reaction, and the relative expression level of each miRNA to 5S snRNA was evaluated using 2^−(CTmiRNA−CT5SRNA)^ method. The expression level of miRNA in ovary of Anhuai goats in Fols and Luts was determined individually. The primers of miRNAs for qRT-PCR were shown in Table 1.

**Table 1:** Summary of miRNA primers sequences for the RT-PCR

Name	Sequence (5′ → 3′)	Length (nt)	GC (%)
chi-Let-7f-5p	TGAGGTAGTAGATTGTATAGTT	22	31.82
chi-miR-21-5p	TAGCTTATCAGACTGATGTTGAC	23	39.13
chi-miR-10a-5p	TACCCTGTAGATCCGAATTTGT	22	40.91
chi-miR-22-3p	AAGCTGCCAGTTGAAGAAC	19	47.37
chi-miR-125a-5p	TCCCTGAGACCCTTTAACCTGT	22	50.00

## Result

### Basic analysis of data

In total, 11,727,490 and 12,209,928 raw reads were obtained from the ovaries of Anhuai goats in Fols and Luts, respectively. After eliminating low-quality sequences and removing the sequences shorter than 18 nt, reads without 3′ ligation and insert tags, contaminants formed by adapter–adapter ligation, ultimately, 11,546,193 and 12,013,471 clean reads, which accounted for 98.78 and 98.79 % of the total sequence reads, were obtained from the ovaries of Anhuai goats in Fols and Luts, respectively. The clean reads were used for further analysis (Table 2).

**Table 2:** The classification of total small RNA tags by Solexa sequencing

Type	Follicular phase		Luteal phase	
	Count	%	Count	%
Total_reads	11,727,490		12,209,928	
High_quality	11,688,783	100	12,160,407	100
3′adapter_null	44,810	0.38	50,439	0.41
Insert_null	362	0.01	687	0.01
5′adapter_contaminants	3676	0.03	2498	0.02
Smaller_than_18nt	93,733	0.80	93,268	0.77
polyA	9	0.00	44	0.00
Clean_reads	11,546,193	98.78	12,013,471	98.79

**Fig. 1:**
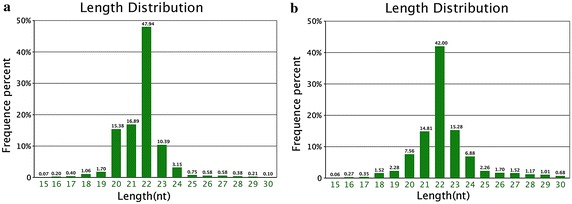
Distribution of sequence lengths of the sequencing results in **a** FO and **b** LO library

Length distribution of tags was showed in Fig. 1, where the horizontal coordinates were tags lengths and the vertical coordinates were percent of tags. The majority of the sRNA tags were within 20–24 nt. The typical size of Dicer-derived products and peaked at the distribution length was the sequences 22 nt in length, and accounted for 47.94 and 42 % of the total sequence reads in the Fols and Luts, respectively. The length distribution of the clean reads was similar in the Fols and Luts.

**Fig. 2:**
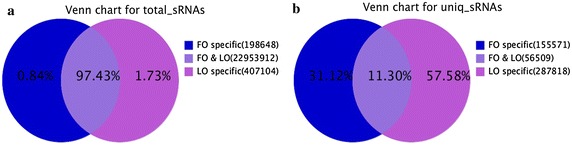
Number of **a** total and **b** unique sRNA tags between the two libraries

In total, 22,953,912 common sequences were obtained in Fols and Luts, accounted for 97.43 % of the total sequence reads in the two libraries. In addition, 155,571 (31.12 %) and 287,818 (57.58 %) specific sequences were obtained from Fols and Luts, respectively (Fig. 2).

### Standard bioinformatics analysis

Tags were mapped to genome by SOAP or bowtie to analyze hircine gene expression and distribution in the two libraries. 11,546,193 (78.92 %) and 9,624,248 (80.11 %) clean reads in Fols and Luts were mapped to the hircine genome, respectively. sRNA was mapped to repeat with processes, screen and remove repeat associated tags. We found that the number of unique sRNA tags mapped to repeat associated sequence Ambi was the highest, and the number of total sRNA tags mapped to total associated repeats SINE/SS was the highest in Fols. The results of genome mapping and repeat reads in the Luts were similar to the Fols.

**Fig. 3:**
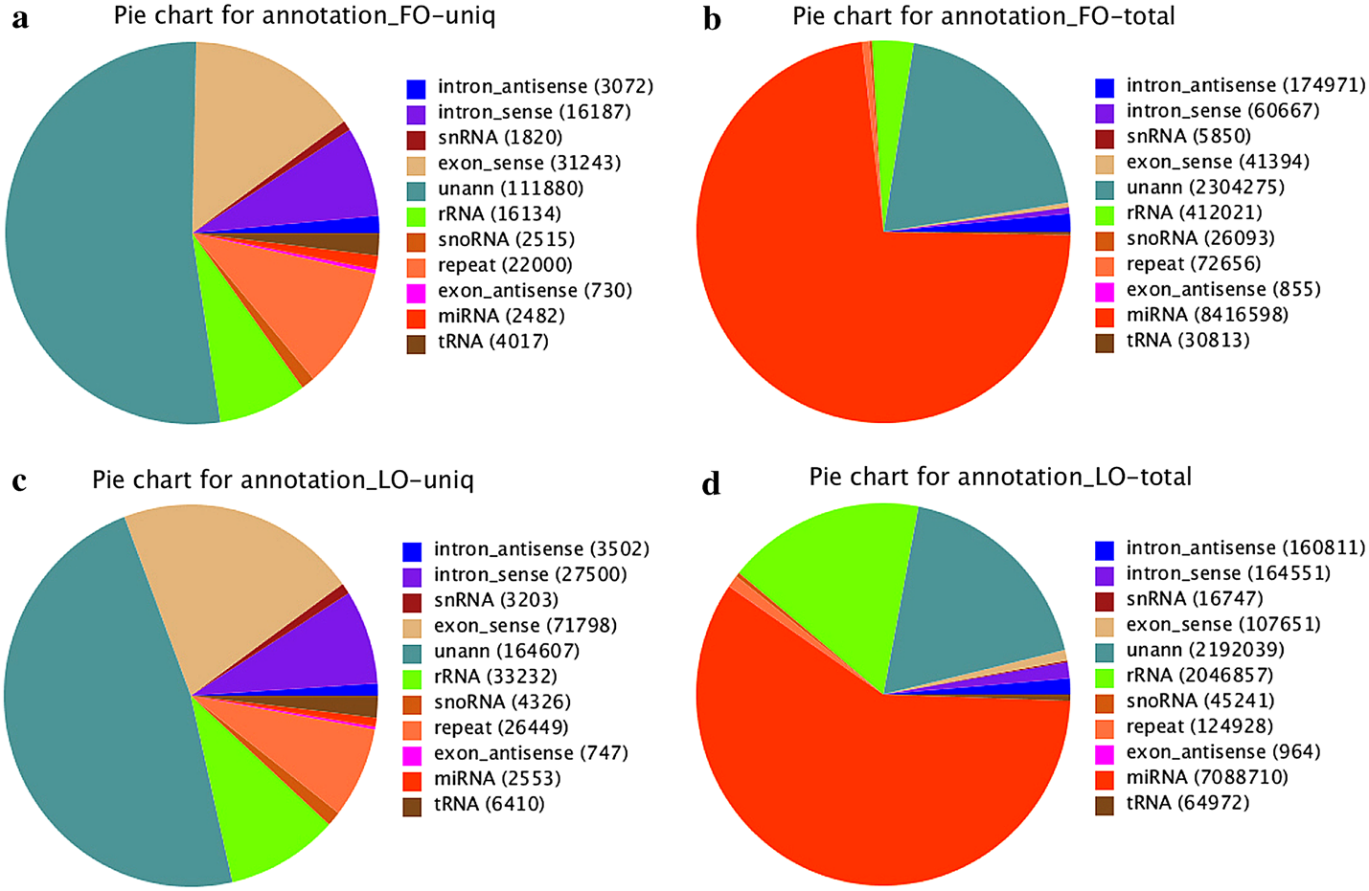
Composition of small RNA classes of the sequencing. (Note: **a** Total number of unique sequences in the FO library. **b** Total number of reads in the FO library. **c** Total number of unique sequences in the LO library. **d** Total number of reads in the LO library.)

Totally, 212,080 and 344,327 clean sequences were obtained from Fols and Luts, respectively. The proportion of total rRNA was 3.57 and 17.04 % in Fols and Luts, respectively, which meant that the collected ovaries samples were of high quality. In the Fols and Luts, miRNAs have 8,416,598 (72.90 %) and 7,088,710 (59.01 %) of the clean reads, and 1.17 and 0.74 % of the unique reads (Fig. 3). The results of the two libraries showed that the majority of total reads was classified as miRNA, but unann, which was the sRNA which hadn’t mapped to any annotation (rRNAetc, known miRNA, piRNA, repeat, exon, intron), comprised the highest fraction of the unique reads.

### Known miRNA and novel miRNA analysis

Slight mismatches between sequences were considered, 342 and 352 known miRNAs were identified in the Fols and Luts. Among them, 320 known miRNAs were co-expressed, 19 and 33 miRNAs were specifically expressed in the Fols and Luts, respectively. And 70 and 94 novel miRNAs were identified in the Fols and Luts libraries, in which 25 novel miRNAs were co-expressed, 45 and 69 miRNAs were specifically expressed in the Fols and Luts, respectively. Novel miRNA basic information including the location and structure of the goat genome was listed in Additional files 1 and 2.

### Differentially expressed miRNAs between the two libraries

To detect the Fols–Luts transition related genes, the expression levels of all detected miRNAs from Fols and Luts were calculated using the reads per kilo bases per million reads (RPKM) method. According to the fold-change calculations, the expression levels of 125 known miRNAs and 51 novel miRNAs were different between the Fols and Luts. Totally, 30.91 % of the known miRNAs were extremely and significantly different between the two libraries (p < 0.01), and 2.69 % of the known miRNAs were significantly different (0.01 ≤ p < 0.05). In addition, 40.34 % of the novel miRNAs were extremely and significantly different (p < 0.01), and 2.52 % of the novel miRNAs were significantly different (0.01 ≤ p < 0.05). Only 4 known miRNAs were specific expressed (p < 0.05) in the Luts.

**Fig. 4:**
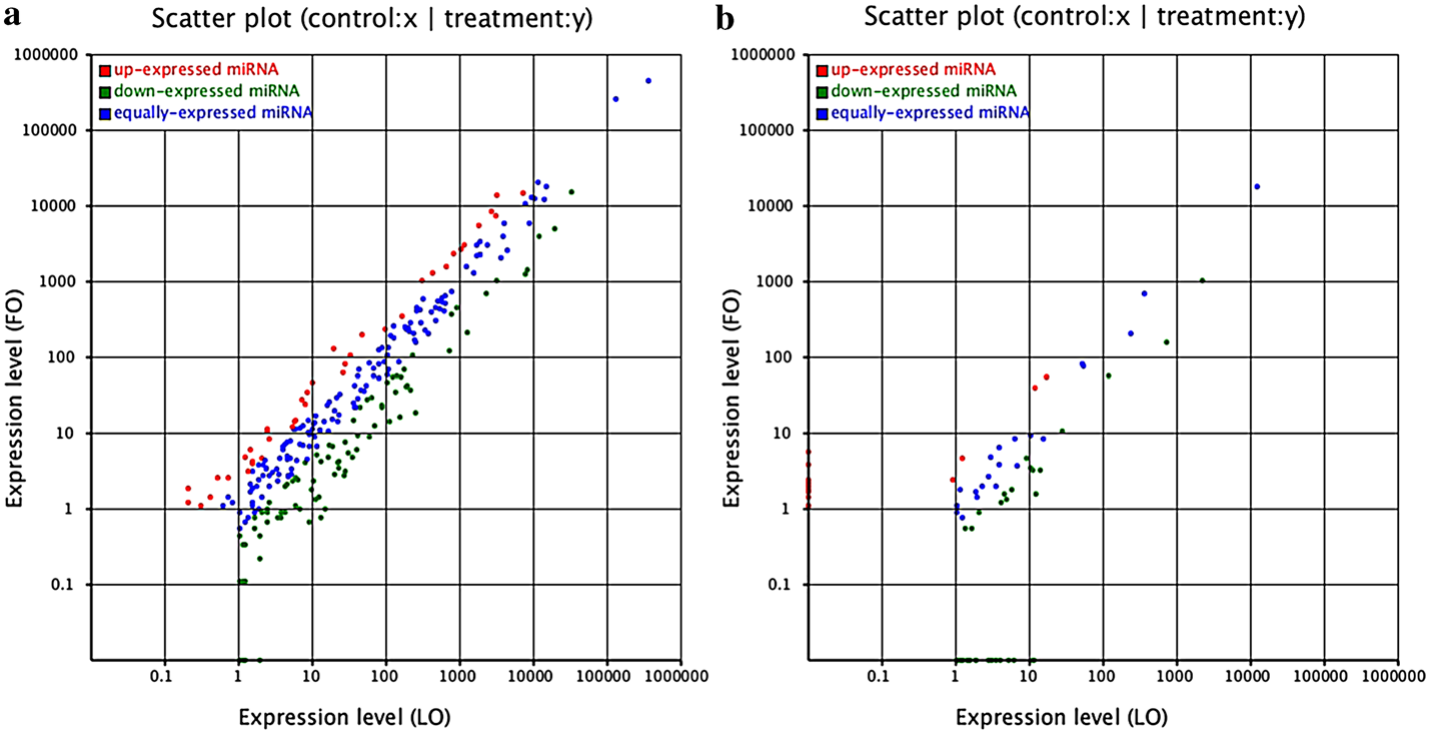
Differential expression analysis of **a** known and **b** novel miRNA between the two libraries. (Note: The scatter plot of differentially expressed miRNAs (LO: *X*-*axis*, FO: *Y*-*axis*). The X and Y show the expression level of miRNAs in the two samples respectively. *Red points* represent miRNAs with ratio >2; *Blue* *points* represent miRNAs with 1/2 <ratio ≤2; *Green points* represent miRNAs with ratio ≤1/2. Ratio = normalized expression of the treatment/normalized expression of the control.)

Let-7f, miR-125a and miR-21 were the top three differentially expressed known miRNAs between the two libraries. Let-7b and miR-125a can promote angiogenesis in the corpus luteum, and miR-21 can promote the survival of follicular cells during ovulation (Newcomb et al. 2015). It was inferred that miR-21, let-7f, miR-125a may be related to Fols-Luts transition in Anhuai goats. The differentially expressed miRNAs between the two libraries were showed in Fig. 4 and Additional files 3 and 4. Ten and Twenty-two novel miRNAs were specific expressed and significantly different (p < 0.05) in the Fols and Luts, respectively. The expression of the specifically expressed miRNAs were very low (<5), there were only 2 and 3 specific expressed novel miRNAs were higher than 1000 in Fols and Luts, respectively (Tables 3, 4).

**Table 3:** Expressed level of higher than 1000 and significant differently in the known miRNAs

Follicular phase			Luteal phase		
miR_name	FO-expressed	FO-std	miR_name	LO-expressed	LO-std
chi-let-7f-5p	135,763	14,899.79	chi-let-7f-5p	318,048	33,046.53
chi-miR-125a-5p	131,983	14,484.94	chi-miR-21-5p	188,869	19,624.29
chi-miR-10a-5p	123,919	13,599.93	chi-miR-22-3p	117,022	12,159.08
chi-miR-99a-5p	77,111	8462.817	chi-miR-127-3p	80,135	8326.365
chi-miR-125b-5p	66,771	7328.018	chi-miR-1468-5p	76,409	7939.218
chi-let-7c-5p	49,556	5438.697	chi-miR-125a-5p	69,394	7210.33
chi-miR-21-5p	44,691	4904.771	chi-miR-10a-5p	31,274	3249.501
chi-miR-22-3p	35,658	3913.412	chi-miR-126-5p	30,474	3166.377
chi-miR-192-5p	27,251	2990.757	chi-miR-125b-5p	30,389	3157.545
chi-miR-25-3p	24,413	2679.29	chi-miR-99a-5p	25,881	2689.145
chi-miR-101-3p	20,817	2284.635	chi-miR-411a-5p	22,277	2314.674
chi-miR-125b-3p	13,989	1535.272	chi-let-7c-5p	17,466	1814.791
chi-miR-127-3p	12,700	1393.806	chi-miR-409-3p	12,268	1274.697
chi-miR-130a-3p	11,691	1283.07	chi-miR-192-5p	11,207	1164.455
chi-miR-1468-5p	11,266	1236.427	chi-miR-25-3p	10,116	1051.095

**Table 4:** Expressed level of higher than 1000 and significant differently in the novel miRNAs

Follicular phase			Luteal phase		
miR_name	FO-expressed	FO-std	miR_name	LO-expressed	LO-std
novel_mir_159	9292	1019.783	novel_mir_159	21,361	2219.498
novel_mir_55	1411	154.8551	novel_mir_55	7109	738.6551
			novel_mir_176	1139	118.3469

Cluster miRNA with similar expression pattern was showed in the Additional file 4: Figure S1, Additional file 5: Figure S2, in which red indicated that the miRNA has higher expression level in the Fols, green indicated that the miRNA has higher expression in Luts and gray indicated that the miRNA has no expression at least one sample.

### Gene ontology (GO) enrichment analysis of target genes of differentially expressed miRNAs

We used RNA hybrid software to predict the target gene of significantly differentially expressed miRNAs by searching the hircine reference gene database. For the known miRNAs and novel miRNAs library, 3420 and 4266 target genes of differentially expressed miRNAs were identified, respectively.

Known miRNA GO enrichment for gene background based on the cellular component showed that 1105 target genes were mapped to GO terms in the database. Compared to the reference gene background, 6 GO terms, actin filament bundle, protein-lipid complex, plasma lipoprotein particle, mitochondrial proton-transporting ATP synthase complex, proton-transporting ATP synthase complex, and extracellular space, were significantly enriched. Moreover, 1056 target genes were assigned different functions in the molecular function. Compared to the reference gene background, 1 GO term, lipase activity, was significantly enriched. In total, 1035 target genes were assigned biological process based on gene background. Compared to the reference gene background, 5 GO terms, negative regulation of endocytosis, regulation of lipoprotein oxidation, negative regulation of lipoprotein oxidation, regulation of lipoprotein metabolic process, negative regulation of lipoprotein metabolic process, were significantly enriched.

**Fig. 5:**
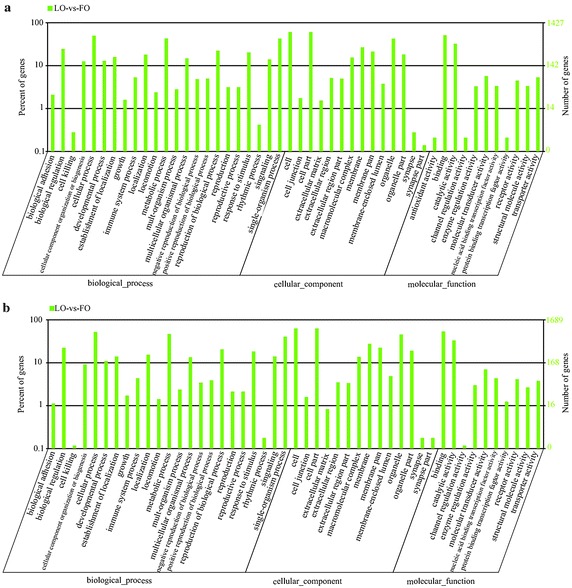
GO classification annotated for the target gene of miRNA. **a** GO classification annotated for the target gene of known miRNA LO-vs-FO. **b** GO classification annotated for the target gene of novel miRNA LO-vs-FO

Novel miRNA GO enrichment for gene background based on the cellular component, molecular function, biological process were the same as known miRNA. 1311 target genes were mapped to the cellular component. Compared to the reference gene background, 3 GO terms, late endosome, basal plasma membrane, basal part of cell, were significantly enriched. Molecular function analysis showed that 1307 target genes were assigned to different functions. In comparison with the reference gene background, 1 GO term, nucleobase-containing compound transmembrane transporter activity, was significantly enriched. In addition, 1225 target genes were related to biological processes, and compared to the reference gene background, only 2 GO terms, cytokine biosynthetic process, cytokine metabolic process, were significantly enriched (Fig. 5; Additional files 5, 6). We found that the mapped maximum number is cell term in cellular component. The cellular process and binding was the maximum number in biological process and molecular function, respectively.

### KEGG pathway analysis of target genes of differentially expressed miRNAs

KEGG pathway annotation analysis of the background genes revealed 2298 target genes of differentially expressed known miRNAs were annotated to the relevant biological functions. However, KEGG pathway analysis of the target genes revealed that no pathways were significantly enriched (Q value <0.05). Moreover, 2881 target genes of novel miRNAs were annotated to the relevant biological functions, and 10 pathways were significantly (Q value <0.05) enriched (Additional files 7, 8), which are GnRH signaling pathway, Fat digestion and absorption, Glycine, serine and threonine metabolism, Tyrosine metabolism, Styrene degradation, Osteoclast differentiation, Caffeine metabolism, alpha-linolenic acid metabolism, Fc epsilon RI signaling pathway, and melanogenesis The differentially expressed novel miRNAs which participated the pathways may be related to Fol-Lut transition of Anhuai goat.

## Discussion

Goats’ total productivity determined their economic efficiency, and the productivity is more dependent on fertility and prolificacy of the female goats (Mascarenhas et al. 1995; Khanum et al. 2007; Zhang et al. 2009). However, hircine fecundity is relatively low (Zhang et al. 2013), and the traditional breeding technology was inefficient. Therefore, molecular assisted breeding technology was thrived in the recent years (Huang et al. 2011; McBride et al. 2012), and miRNAs which could regulate mRNA degradation at the post-transcriptional level or inhibit mRNA translation to modulate gene expression, provided a new method for molecular assisted breed. It has been showed that miR-290-295 and miR-17-92 can promote the progression of cell cycle, the differentiation of primordial germ cells (PGCs), meanwhile, the process differentiation of PGCs gradually reduced the expression of miR-141, miR-200a, miR-200c and miR-323 (Galloway et al. 2000). The survival and migration of primordial germ cells were related of to miR-290-295 family. The mission of miR-290-295 would lead to the infertility of female mice (Cummins et al. 1983).

To investigate the regulation mechanism of transition of Fols to Lutsin goats at post-transcriptional level, we characterized and investigated the expression of miRNAs in the ovaries of Anhuai goats in follicular (Fols) and luteal (Luts) phase by deep sequencing technology. It has been showed that, 80 % of miRNA from intron promoter region, and the other approximately 20 % of miRNA from the exons non-coding region, as well as a little part of the genome miRNA from transposon region and repeat of the sequence (Kim and Nam 2006; Kim et al. 2009). In the exons and introns annotation, about 80 % of sequences were mapped to introns in the two libraries, while the proportion of the sequence aligned to repeat sequences was very low, which was same to researches carried by predecessors.

Compared to the other studies (Yuan et al. 2013), the number of sRNA obtained in the study was relatively less. 339 and 353 known miRNAs, and 80 and 113 novel miRNAs were identified in the Fols and Luts libraries. However, the goat genome which published in the miRBase 21.0 database in June 2014, was used as reference in the study. And 436 mature sequences and 267 precursor sequences were added in the goat genome in miRBase 21.0. Compared to all animals miRNAs, the objective reference improve the accuracy of prediction.

Currently researches about the development regulation of follicular miRNA has made advancement, and a number of functions miRNA in the development of luteum were identified, such as expression of miR-125b in granulosa cells, ensheathing cells and corpus luteum, which target gene is LIF, the expression of miR-145 in sheath cells and luteum, which target gene is CDKN1A, and expression of miR-199a-3pin sheath cells and luteal, which target gene is PTGS2 (McBride et al. 2012). miR-21 could inhibit apoptosis of factor promoting survival of follicles and follicular transition to luteal (Easow et al. 2007; Kanehisa et al. 2008). To investigate the regulation mechanisms of follicular to luteal transition at the post-transcriptional level in the Anhuai goat, the differentially expressed miRNA were analyzed in the two constructed libraries in this study. We found that the specifically expressed miRNAs could be involved in the regulation of the relevant pathways. Althrough expression level of the specifically expressed miRNAs was relatively low.

The top three highest differentially expressed miRNA in two libraries was let-7f, miR-21 and miR-125a. The expression of let-7f in the Fols was significantly higher than which in the Luts. miRNAs let-7a, let-7b, let-7c, and let-7i were significantly decreased in early atretic and progressively atretic porcine ovary follicles compared with healthy follicles, while let-7g was highly expressed during follicle atresia (Cao et al. 2015). Let-7b has been identified in the development of normal luteum which was involved in FSH secretion regulation (Ye et al. 2013). Let-7b can promote angiogenesis in the corpus luteum, but estrogen and progesterone reduced will lead to expression of let-7f reduced in mice, which target gene is Timp1 (Newcomb et al. 2015).

In the study, the expression of miR-21 in the Luts was significantly higher than which in the Fols, while, the expression of miR-125a in the Fols was significantly higher than which in the Luts. miR-21 can promote the survival of follicular cells during ovulation (Newcomb et al. 2015). The second peak of FSH stimulates the development of large estrogenic follicles during the early luteal phase, but the period of functional dominance is shorter than the period of morphological dominance (Carlos 1997). miR-125a may regulate vascular endothelial cell growth factor A to affect angiogenesis (Dai et al. 2015). The expression of miR-21, miR-125b, let-7a and let-7b accounted for 40 % of all sequences (McBride et al. 2012). miR-125a and miR-125b were expressed in goat ovarian granulosa cells, ensheathing cells and corpus luteum in the same family, but its function was uncertain. Higher progesterone concentrations may accelerate follicular turnover probably by an early decline of the negative feedback action of the largest follicle of Wave 1. This is followed by an early emergence of Wave 2 (Menchaca and Rubianes 2002). In consideration of let-7f, the function of miR-125a is not clear in follicle and corpus luteum, some further experimentation is needed.

We could obtain a better understanding from the molecular functions, cellular components and biological processes of target genes by GO annotation and KEGG Pathway analyses (Ji et al. 2012). For the target genes of differentially expressed known miRNAs, Lipase activity, regulation of lipoprotein oxidation, negative regulation of lipoprotein oxidation, regulation of lipoprotein metabolic process, negative regulation of lipoprotein metabolic process was significantly enriched. In addition, there were 5 terms were enriched in differentially expressed novel miRNA library (late endosome, basal plasma membrane, basal part of cell, nucleobase-containing compound transmembrane transporter activity, cytokine biosynthetic process, cytokine metabolic process). And functional analysis of the target gene is targeted cell, cellular process and binding, indicating that differentially expressed miRNAs mainly involved in cell activities.

In the KEGG pathway analysis, it was worth to note that GnRH signaling pathway, Fat digestion and absorption, Glycine, serine and threonine metabolism in the significantly enriched pathway in the novel miRNA library. GnRH is released from the hypothalamus and controls the release of the FSH and LH from the pituitary gland which induces ovarian steroid secretion that impacts the hypothalamus and pituitary through a feedback mechanism (Richards and Pangas 2010a). According to research on specific breeds of mice, the roles of bone morphogenetic proteins (BMPs), activins, Smads, Wnt signaling, and aspects of the FSH and LH signaling cascades are emphasized in the regulation of cyclic ovarian activity (Puglisi et al. 2004; Tomizuka et al. 2008). These significantly enriched pathways may be related to follicular to luteal transition in goat ovaries. GO annotation and KEGG Pathway analyses can provide a reference to us for the later research.

## Conclusions

In summary, 320 known miRNAs were co-expressed, 320 known miRNAs were co-expressed in the two phases, 339 and 353 known miRNAs were specifically expressed in the hircine ovaries of Anhuai goats during the follicular and luteal phase libraries, respectively. The highest expressed known miRNA was let-7f in the two libraries, followed by the miR-21, miR-125a in the follicular and the luteal phase libraries, respectively. And 119 novel miRNAs were predicted in total. GnRH signaling pathway, Fat digestion and absorption, Glycine, serine and threonine metabolism were pointed out for significantly enriched in the KEGG pathway analyses. The result may help to further understand the role of miRNAs in the regulation of follicular-luteal transition of goats in the future.

## Additional files

10.1186/s40064-016-2902-1 Novel miRNA basic information about the location and structure of the goat genome in the follicular phase.
10.1186/s40064-016-2902-1 Novel miRNA basic information about the location and structure of the goat genome in luteal phase.
10.1186/s40064-016-2902-1 The differentially expressed known miRNAs between the two libraries.
10.1186/s40064-016-2902-1 The differentially expressed novel miRNAs between the two libraries.
10.1186/s40064-016-2902-1 Clustering analysis of novel miRNAs differentially expressed in the follicular and luteal phase libraries.
10.1186/s40064-016-2902-1 Clustering analysis of known miRNAs differentially expressed in the follicular and luteal phase libraries.
10.1186/s40064-016-2902-1 GO enrichment analysis for the target genes of differentially expressed known miRNAs (LO-vs-FO).
10.1186/s40064-016-2902-1 GO enrichment analysis for the target genes of differentially expressed novel miRNAs (LO-vs-FO).
10.1186/s40064-016-2902-1 KEGG pathways for the target genes of differentially expressed known miRNAs (LO-vs-FO).
10.1186/s40064-016-2902-1 KEGG pathways for the target genes of differentially expressed novel miRNAs (LO-vs-FO).
