# Supplementary figures and images for: Comparative profiling of differentially expressed microRNAs between the follicular and luteal phases ovaries of goats
Source: Springerplus. 2016 Aug 2;5(1):1233. doi: 10.1186/s40064-016-2902-1 (PMC4993730; doi:10.1186/s40064-016-2902-1)

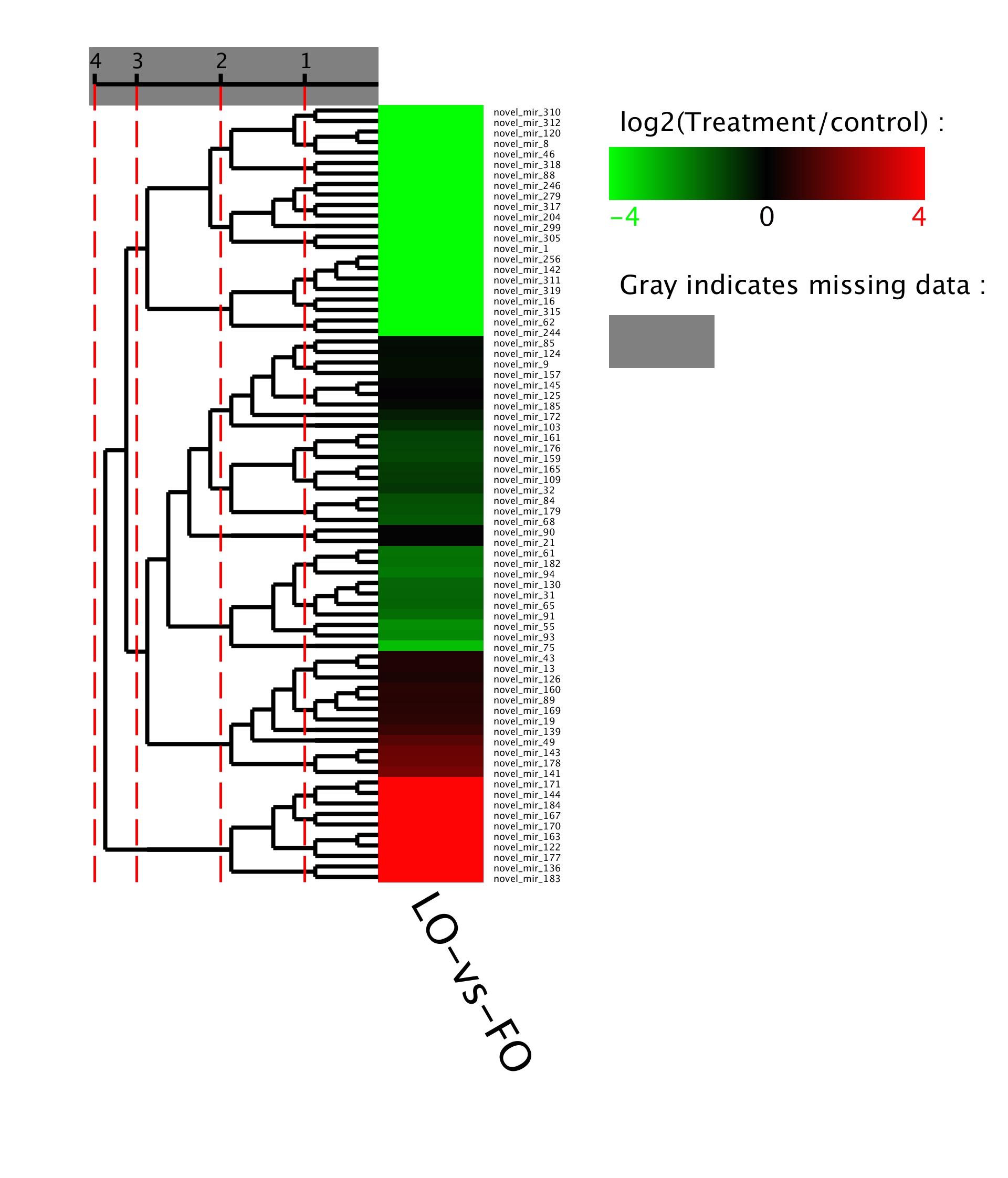

Supplement: Supplementary file 5 — 10.1186/s40064-016-2902-1 Clustering analysis of novel miRNAs differentially expressed in the follicular and luteal phase libraries. [file 40064_2016_2902_MOESM5_ESM.jpg]

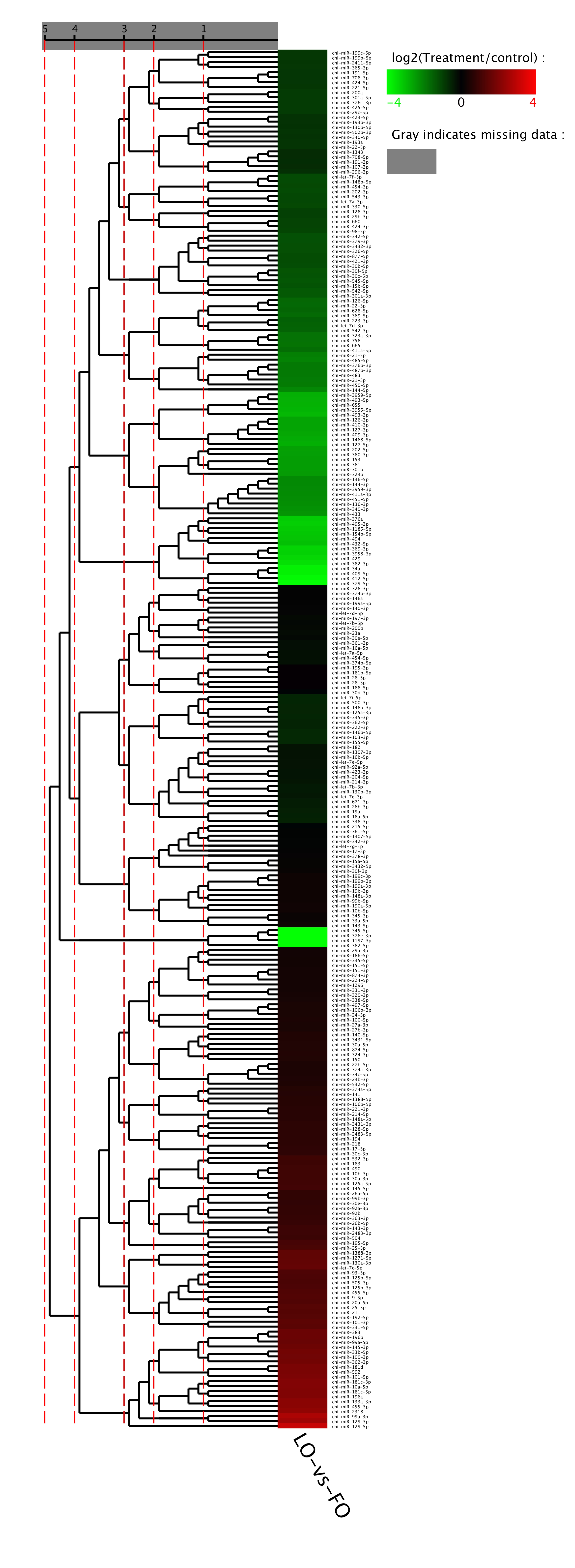

Supplement: Supplementary file 6 — 10.1186/s40064-016-2902-1 Clustering analysis of known miRNAs differentially expressed in the follicular and luteal phase libraries. [file 40064_2016_2902_MOESM6_ESM.jpg]
